# Georgia State Medical Association

**Published:** 1892-05

**Authors:** 


### *SOCIETY REPORTS.*

---

### GEORGIA STATE MEDICAL ASSOCIATION.

---

*Forty-Third Annual Meeting, held at Columbus, April* 20, 21, *and* 22, 1892.

---

### FIRST DAY—MORNING SESSION.

The Association convened in Springer's Opera House, and was called to order at 11:25 A. M., by the President, Dr. G. W Mulligan, of Washington.

The address of welcome was delivered by the Hon. J. B. Slade, Mayor of Columbus, which was responded to by Dr. J. B. Baird, of Atlanta.

Prayer was then offered by the Rev. Robert Harris.

PRESIDENT MULLIGAN delivered his annual address, in which he pleaded for the abandonment of all unscientific methods of reasoning and investigation, and for a still firmer alliance with those principles which, in other branches of knowledge, have produced such brilliant successes. He pleaded for less reliance on the blind gropings of empiricism and a still closer affiliation with every method of modern science. He also pleaded for the exclusion of all fallacies and the adoption of every safeguard which may indicate a wrong direction in our studies, and for the entire displacement of self in the investigation of life, both in health and disease, and a concentration of all our powers in searching out and fortifying the laws governing the relation and succession of these phenomena.

DR. J. M. SPENCE, of Waresboro, read a short paper entitled

### A CASE OF OVARIAN CYST.

May 1, 1890, he was called in consultation with Dr. W. P. Williams, of Waycross, to see a case. Five years prior to his visit the patient gave birth to twins, attended, however, with great difficulty. An undergraduate attended her. Her health was apparently good until she became pregnant again, twelve months hence. The issue of the second pregnancy was triplets, the patient again enduring a state of prolonged labor and excruciating pain, under the supervision of the same quack. In about two months something began to grow in the region of the right ovary. It grew rapidly for about three years. After careful examination an exploring needle was introduced, which, on its withdrawal, was found to contain a thick, straw-colored fluid, about the consistency of ordinary syrup. The legs and whole lower extremities were infiltrated with water, her tongue coated, and bowels costive—the infiltration being caused by compression of the tumor on the kidneys and ureter. They next took a measurement of the abdomen, which was six and a half feet, and from the pubes to the ensiform appendix the distance was three feet and nine inches. Calomel, pulverized rhubarb, carbonate of soda in repeated doses, together with iron, strychnine and digitalis, were given for a few days. Six days later they met and introduced a trocar and canula through the walls of the abdomen, and through the aperture thus made there came seventy quarts (which seems almost incredible) of a thick straw-colored fluid. The same treatment was continued at their second visit, two weeks later, and at this time twenty-eight quarts of a similar fluid were withdrawn, together with something that looked like human brains. At a third visit, two weeks later, they drew thirty quarts of similar fluid, but the trocar would frequently become choked with the brain-looking matter discovered after the operation. Ten days later the patient was seized with cholera morbus and died.

DR. D. C. HURT, of Columbus, followed with a paper on

### THE INFLUENCE OF CIVILIZATION AND SOCIAL LIFE UPON WOMEN, AS VIEWED FROM A MEDICAL STANDPOINT.

He said that while in the present day so much attention is being paid to the diseases peculiar to women, it is highly proper that we, as medical men, should dwell with serious thoughts upon the causes and conditions that bring about these results.

It is the office of the physician to assiduously apply himself (1) to prevent, and (2) to remove, as far as possible, all maladies that human flesh is heir to ; but when we open our eyes fully to the conditions of thirty per cent. of our women of the present day, the facts stare us in the face that these evils are wonderfully multiplied.

A practical demonstration exists to-day by comparison of our savage and civilized women in America. While the Indian squaw is unacquainted with female diseases and broken-down constitutions on account thereof, our cultivated and civilized women are growing more and more intolerant of that life which pertains to their special duties as females. The Indian squaw lives in her hut and performs manual duties and undergoes hard physical labor without any interference with her female nature, and to her the act of parturition is looked upon without dread, carries with it but little pain, and scarcely interrupts her duties from day to day. Our civilized and cultured women, if forsooth they arrive at healthy womanhood and maturity, are liable at each turn of life to become a prey to some one or more of the thousand maladies that eventually bring them under the care of the gynecologist. Why this change ? The style of dress which exposes the body to the most inclement weather, producing reversions of cold and heat ; the waist being tightly laced by steels, the body being kept in such a state of contortion that deformities result and produce deleterious effects upon parturient women, as well as disturb the functions of the whole organism.

With our present knowledge of antiseptics we destroy those bacteria which lie broadcast about our homes, and arrest the germ-producing process already at work in the consumption of our bodies ; and we should with equal dexterity and confidence in our skill remove the cess-pools of moral corruption and elevate the mind to higher functions and duties of life-making, avoiding the reversions of sin which pander to depraved appetites, thereby guarding us against those depravities of our nature which tend to foster in us vitiated blood, whose domain it is to contaminate our whole being. We should guard our commonwealth against the concealed viper discovered only by medical men, which lies ever and anon in our pathway seeking anxiously every opportunity of injecting its venom into the moral and social veins of human life, thereby leaving in our systems that poison which is to be handed to our posterity as a defiled inheritance.

Adjourned.

---

### FIRST DAY—AFTERNOON SESSION.

The Association reassembled at 3 P. M. The first paper read was by Dr. M. B. Hutchins, of Atlanta, entitled

### THE RELATION BETWEEN SKIN DISEASES AND THE GENERAL HEALTH.

He dwelt principally upon eczema, because it is the best known and most common skin disease, comprising about one-third of all cases. We find nearly every type of skin disease is one or the other form of eczema; and so what applies in general to eczema applies to all the others. Two things were evident after reading the opinions of the various writers upon skin diseases :

1. We can be positive that all skin diseases are not "blood diseases," or the idea would have been universally accepted.

2. Their disagreement in some details and their general unanimity in other details shows that there is as yet much to be learned concerning the actual etiology of skin diseases.

The germ theory received scant attention in the paper, because the discoveries of skin disease producing germs are as yet in a most decided condition of infancy, and the discussion of it would lead us farther into the dark than the necessities of the case demand.

### THE TREATMENT OF ABORTION AND SOME OF THE COMPLICATIONS INCIDENT THERETO.

DR. W. A. CROW, of Atlanta, read a paper on this subject. He emphasized the following points :

1. The marked tendency to infection in all cases of abortion.

2. The urgent necessity of removing, as far as possible, all sources of infection. This includes clean hands, clean instruments, cleanliness of person and bedding.

3. The importance of thoroughly satisfying ourselves in all cases of the complete removal of all the fœtal membranes and placental tissue.

4. Keep the patient under observation for at least two months, with the most favorable surroundings in order to complete involution of the enlarged uterus, building up the general health and keeping the bowels open, if necessary, with salines.

5. Be urgent relative to advice in avoiding the possibility of becoming pregnant for at least six months, as sexual connections produce more or less irritation and congestion of the parts and thereby retard, if not in a measure stop, the condition we most desire in these cases, the normal restoration of the parts.

DR. H. PERDUE, of Barnesville, read a paper on

THE TREATMENT OF PNEUMONIA WITH REPORT OF CASES.

Since the first of January, he had had, besides several of the lobular variety, fifteen cases of the lobar. Three of the lobar class had one lobe of the lungs affected, four had two, seven had three, and one had four. This shows an unusual *pro rata* number of lobes attacked, yet he had not lost a single case.

In lobar pneumonia he uses peroxide of hydrogen with good effect. It is given for its germicidal properties; the oxygen it furnished the patients exhilarated and strengthened them. If it nauseates—and sometimes it does—the quantity should be diminished. He feels confident it has saved valuable lives. Throughout the stages of congestion and consolidation he gives phenacetine in five grain doses in connection with whiskey or milk punch every four hours, unless the temperature should get below 103 F. Good nursing is an important factor in the management of pneumonia patients. A nutritious liquid diet, such as sweet milk, beef tea and palatable broths should be given. In convalescence stimulating expectorants and tonics are indicated.

In catarrhal pneumonia the supporting treatment should be given from the beginning. He generally gives carbonate or muriate of ammonia during the entire treatment. Quinine he considers a valuable remedy in reducing temperature, and as an aid to resolution. Mild mustard poultices or blisters to the chest are valuable; also flannel cloths or jackets saturated with turpentine, diluted with oil if the patient is a child. Great care should be exercised in convalescence to prevent a relapse or a second attack.

### WHAT IS GYNECOLOGY?

DR. R. R. KIME, of Atlanta, read this paper. He briefly reviewed the progress made in gynecology, and said the Father of Medicine recognized the sympathy existing between the mammary gland and the uterus, which he utilized by cupping them for uterine hemorrhage.

Another twelve centuries brought us to the nineteenth century in which fads, fashions and cycles held sway as in the olden times. To illustrate: we have only to mention that a few years since everybody slit or divided the cervix; a few years later everybody sewed it up, and now everybody divulses or dilates, and drains.

A few years since everybody probed or sounded the uterus, applied caustics to ulcers of the os. Now the sound is not often used and ulcers of the os are a nonentity. Within the last three or four decades the advancement in gynecology has been so rapid that it is difficult to keep in the line of march as the procession advances to new thoughts and new discoveries. Ovariotomy is about the only operation that has not been greatly abused or oft resorted to without just provocation. It first had met with violent opposition, but to-day stands as an established procedure. The improved technique with lessened mortality are about the only improvements since the operation was first performed. The speaker was glad to see gynecology as fast approaching a scientific basis. In the last two decades it has outstripped all other specialties of medicine in new discoveries and improved operative technique, thereby saving many of "God's noblest gift, the woman perfected."

He concluded by saying that the South had not been asleep, that she furnishes and has furnished her quota of active original workers in this speciality, that the Southern Surgical and Gynecological Association is maintained within her borders, and among her sons may be found such names as McDowell, Sims, Taliaferro and Battey, with many other eminent active workers in that line.

Adjourned.

---

SECOND DAY—MORNING SESSION, APRIL 21ST.

DR. A. C. BLAIN, of Macon, read a paper on

REMITTENT FEVER.

In persons exposed to the malarial miasm it is a good plan to give daily doses of quinine (five to ten grains), each morning, and they should avoid night exposure. Should the practitioner see the patient in the prodromic stage of lassitude, furred tongue, foul breath, he believes that he can often prevent an attack of remittent fever by giving a mercurial cathartic, followed by quinine and a mineral acid.

*Actual treatment.*—The patient should be put to bed in a well ventilated room. Patients require good nursing. Regulate diet; a fluid diet is necessary and milk is the best to rely upon. The practitioner may have to boil the milk and give it iced, or he may peptonize it when necessary. He believes it best to give all nourishment in small quantities and at frequent intervals. In excessive temperature he relies upon phenacetine as the best drug at his command, in doses of from five to ten grains, as the case may demand. The pain in the limbs during convalescence can be controlled by phenacetine and salol. Convalescence will be greatly assisted by a tonic of nux vomica combined with nitro-muriatic acid and some one of the bitters.

DR. W. E. B. DAVIS, of Rome, read a paper entitled

THE SURGICAL TREATMENT OF PUERPERAL PERITONITIS.

He said that puerperal fever was no longer regarded as a distinct affection, a disease *sui generis*, but is looked upon as a septic fever resulting from infection during the puerperal state. When quarts and gallons of pus are reported as having been removed from the general peritoneal cavity and recovery followed, he believes that the pus has usually resulted from a local collection which has ruptured into the general cavity, and the operation has been done before sufficient time has elapsed for this amount of pus to result from the septic inflammatory process in the general cavity. It is easy to understand how a gallon of pus, which has been shut off from the general cavity by inflammatory exudations and adhesions, and which has only recently ruptured into the peritoneal cavity, can be removed and recovery follow, and it is not difficult, Dr. Davis thinks, to comprehend how this condition might be mistaken for acute, general suppurative peritonitis with a gallon or quart of pus as a result in the cavity, as the pus by its irritating properties will produce an inflammation which would be misleading ; but the condition is quite different from what would be had if the pus had been the result of a general inflammation. Dr. Davis maintains that localized puerperal peritonitis with pus formation is a form of the disease that is amenable to surgical treatment. He had operated on cases where there seemed to be no hope of recovery, with favorable results. He had seen cases, where the pulse was 135 get well after an operation for the relief of this condition.

Frequently the fever from puerperal infection may continue for weeks where there is no pus formation, and in which the ovaries are greatly enlarged and the broad ligament thickened with occlusion of the tubes. He had operated on such a case where the tissues were so soft that his ligatures would cut through, and it was necessary to take up the vessels and ligate them separately. The ovary in this condition is as septic as if it contained pus. Frequently the masses felt in the pelvis following delivery or abortion are due to pelvic peritonitis with adhesions to the intestine and omentum, and when an operation is made to evacuate pus we will often find that in the place of pus this condition present, but where fever is kept up and the patient's condition shows sepsis, it is wiser to open the abdomen, break up the adhesions, and remove the diseased appendages, which are keeping up the infection.

### HOW SHALL WE MANAGE THE UTERUS AFTER ABORTION?

This was the title of a paper read by Dr. K. P. Moore, of Macon. He emphasized the necessity of clearing out the cavity of the uterus in cases of abortion, and by so doing it would be the means of preventing some of the troubles with which the gynecologist has at present to deal.

Dr. C. D. Roy, of Atlanta, followed with a paper on

### COUGH—SOME OF ITS CAUSES AND TREATMENT.

After enumerating and dwelling upon the causes of cough, he touched upon the treatment. Every source of possible irritation should be found and removed. Hypertrophies were best removed with the galvano-cautery, and polypi should be thoroughly extirpated with the wire ecraseur. All septum deviations and spurs, when prominent enough to be a source of irritation, should be removed by whatever method the operator chooses.

The speaker called attention to a cough which is sometimes present about the age of puberty, seemingly dependent upon the condition of the reproductive organs or that of the blood. In the *Lancet* of December, 1890, Sir Andrew Clark cited several cases of this condition occurring in his practice both in boys and girls. Dr. Leaming, of New York, who has since called attention to this subject, has reported such a condition occurring in a young girl, suffering with chlorosis following an attack of mountain fever. The late Sir Morell Mackenzie has also reported such a case. That there is a close relationship between certain conditions of the reproductive organs and certain nervous phenomena in other portions of the body, no one will deny, for day by day the correlation of our bodily functions is growing in importance. Dr. John Mackenzie, of Baltimore, has called attention to certain nasal conditions co-existing with a peculiar state of the reproductive organs, which from their constancy do not seem to be entirely fortuitous. In all such cases the treatment must be general, the aim of the physician being to restore the body to as normal condition as possible by rectifying all morbid states in individual organs.

Adjourned.

### SECOND DAY—AFTERNOON SESSION.

DR. A. W. CALHOUN, of Atlanta, read a paper entitled

### SOME OBSERVATIONS UPON CATARACT OPERATIONS AND AFTER TREATMENT.

He reported 904 operations for cataract. The records of his first forty-seven cases had been lost, so that the report simply embraced the records of 849 cases. Of this number 203 were cases of soft cataract in children, and 654 cases of hard senile cataract in adults. The ages of the children ranged from three months to twenty-five years; the ages of the adults ranged as follows: One hundred and thirty-eight from twenty-five to forty; eighty-two from forty to fifty years of age; ninety-eight from fifty to sixty years of age; one hundred and fifty-seven from sixty to seventy; one hundred and four from seventy to eighty years of age; twenty-eight from eighty to ninety years of age; eleven from ninety to ninety-four years of age, making six hundred and fifty-four of hard cataracts, which, including two hundred and three soft cataracts, makes nine hundred and four. In the beginning of his cataract operations, Dr. Calhoun said if he got ninety-five per cent. of successes he thought he was doing well; but since the injection of cocaine and the advent of antiseptic surgery a hundred per cent. of successes has been the result in his cases.

### GUNSHOT WOUND OF THE STOMACH, WITH REPORT OF A CASE.

DR. A. A. SMITH, of Hawkinsville, read this paper. The patient, a negro, had received a pistol shot said to be in the stomach. After careful examination the doctor found that in the fight, which had occurred two hours previously, his patient had received a wound directly over the stomach. A casual examination with the eye alone was all that was necessary to show that the ball had penetrated the cavity of that organ. A more thorough examination was made with the probe, and it was found that the ball had entered at a point about two inches below and to the left at the tip of the ensiform cartilage of the sternum. The probe passed readily into the cavity of the stomach, and the doctor supposed that the ball had passed through and lodged in some other portion of the body, and did not pursue the examination further. At a third visit, which was about forty-eight hours after the boy had received the shot, he ascertained that there had been two or more free evacuations from the bowels, and to his great surprise the ball had passed with one of the evacuations. The wound healed by first intention, and just one week from the date of the injury the boy returned to his work.

### SOME PRACTICAL POINTS IN REFERENCE TO EYE STRAINS.

This was the title of a paper read by Dr. R. O. Cotter, of Macon.

He was impressed with the observation, that the vast majority of general practitioners overlook the easily substantiated fact that such a great multitude of morbid reflex neuroses are brought about by eye strain; that is to say, uncorrected errors of refraction. He referred especially to hyperopia and hyperopic astigmatism. Myopes generally complain of not being able to see well enough at a distance. Headache was by far the most common accompaniment of hyperopia and astigmatism. These headaches were most common in the temples, frontal region, and vertex. They frequently appeared to be what are known as sick or bilious headaches, and are in any case truly distressing in character. Correcting refractive errors was the most prominent and by far the most scientific part of an oculist's work, and he feared that many practitioners overlooked these cases; that they met with one hundred of such sufferers where they saw one case of cataract.

DR. WILLIS F. WESTMORELAND read a paper on

### PLASTER OF PARIS IN SURGERY.

The object of the paper was not to discuss the various appliances adopted by different surgeons, but to present what he regarded as the best plan—one that showed results in every particular more satisfactory to the surgeon and patient than any other plan that has been presented in the past or present. It was not every plaster of Paris splint (so-called) that is applied to a fractured bone that does well. It must be properly applied, of appropriate material, with the fragments of the fractured bone properly adjusted, to give the results which are so much to be desired. For a time this splint was used only in simple fractures, but now surgeons use them in compound comminuted fractures with the very best results, often saving a life or a limb that was formerly sacrificed to the knife, or other plans of treatment that resulted in the death of the patient. Dr. Westmoreland then gave the details of its application.

Adjourned.

DR. J. NUNN read a paper entitled

### PRELIMINARY OBSERVATIONS ON THE BEHAVIOR OF IODINE IN THE PRESENCE OF CAMPHOR, MENTHOL, THYMOL, ETC.

*Alcohol.*—If we take a saturated alcoholic solution of iodine, add to it as much camphor as it will dissolve, and its solvent capacity for iodine is immensely increased. In fact, it will be found that of a saturated solution of camphor in alcohol, 125 minims will dissolve fifty grains of iodine.

*Carbolic Acid.*—Liquify a quantity of carbolic acid with a little alcohol (about 1 per cent.), saturate the liquified carbolic acid with iodine ; next saturate this iodo-phenic solution with camphor, of which it will dissolve from 300 to 400 per cent. This camphorated iodo-phenol will now dissolve or appropriate iodine until it becomes a dense syrupy liquid. Or,

*Experiment* 3.—Prepare a saturated solution of camphor in carbolic acid. (This formula, which is original with Dr. Nunn, and has been used by him for many years, is possessed of valuable medicinal properties, and is known locally as pheno-camphique.) The result of the saturation of this solution with iodine will be similar to that obtained in Experiment No. 2.

*Salol.*—If camphor and salol are mixed in proper proportions, a liquid results which also has the power of dissolving iodine in large quantity, a blackish liquid resulting.

*Thymol.*—Thymol liquifies when mixed with camphor, and the resulting fluid is also a powerful solvent of iodine.

*Menthol.*—A mixture of menthol and camphor also liquifies, and in this liquid iodine is very soluble.

THIRD DAY—MORNING SESSION, APRIL 22.

The first paper read was by Dr. L. G. Hardman, of Harmony Grove, entitled

ECLAMPSIA AFTER DELIVERY, WITH REPORT OF CASES, in which he summarized as follows:

1. That eclampsia does frequently occur after delivery.

2. That judging from the cases presented, it occurs for the most part in patients with pyelitis.

3. That when we find pus in the urine of pregnant women and can exclude specific disease of the kidneys, any bladder or urethral disease likely to cause it, and thus feel pretty certain that the pus comes from the kidney ; and if it continues and begins to cause any general disturbance of the health, he believes it is our duty to induce abortion or premature labor and thus save our patient a great risk.

DR. A. G. HOBBS, of Atlanta, followed with a short paper on

SOME REMARKS ON TONSIL EXCISIONS, WITH PRESENTATION AND DESCRIPTION OF NEW INSTRUMENT.

The instruments exhibited were the practical outcome of the speaker's efforts through several years to do away with some of the objections to the instruments most commonly used for excising hypertrophied tonsils, as the ring tonsillotomes, the guillotines, wire loops, etc. He still, however, uses the ring blades in young children and in other cases where the tonsil projects decidedly with a base but little, if at all, larger than the body.

The objection to the guillotine or ringed instruments was, that the latter especially so often leave a margin or bruised tissue, which in many cases ends in slough; and, with neither of them is the operator quite certain of the exact amount of the tonsil he will clip, especially if the attempt is made on a mass that is soft, broad and nodulated. The cold wire snare causes great pain and requires a much longer time, and moreover, it leaves a contused stump unless the whole organ be extirpated, which he never does intentionally. The hot wire, even with the most approved guards, endangers the pillars and is, like, the cold wire, quite a formidable instrument, at least in the mind of the patient.

The blades of the instrument exhibited were curved both on the flat and on the edge and are in pairs, right and left. Either blade may, however, be used on either tonsil, especially if the operator is ambidextrous, by making the second upward or downward according to the curve. But in most cases it is easiest and safest to make the section upward to avoid cutting the base of the tongue ; the flat curve of the blade will prevent the danger to the posterior pillar. The firm hold by the double hook enables the operator to lift the mass of tonsil tissue out of its bed from between the pillars to any extent, even when it is partly overlapped by large pillars. In a total of something over four thousand tonsil excisions, Dr. Hobbs finds himself choosing these instruments now in at least seventy-five per cent. of his operations.

DR. MCFADDEN GASTON, of Atlanta, read a paper entitled

EXTIRPATION OF THE RECTUM FOR CARCINOMA. (See page 129.)

The patient, a white man, aged sixty-two years, was referred to Dr. Gaston by Dr. J. I. Darby, of Columbia, Ala. There was no family history of malignant disease, but his appearance led to the conclusion that there might be a cancerous cachexy. Having had occasion to report two cases of papilloma of the rectum with carcinomatous degeneration, which terminated fatally without operation, he recently advised in a similar case extirpation of the entire mass. This was done without serious consequences on May 30, 1891, and promised a good result, but there was a redevelopment of the induration above with a fatal result.

The patient referred to was received at the Providence Infirmary with rectal trouble. Upon digital examination and exploration with the speculum, there was found to exist an induration and thickening of the submucous tissues extending from an inch within the annus up to the recto-colic junction. The mucous membrane presented a dark congested appearance attended with some oozing of blood. There was a marked constriction of the upper portion of the indurated structure, but admitting of the passage of the point of the index finger by forcible upward pressure, giving the impression that the structure above was not involved in the disease. It was therefore determined after consultation with several colleagues, that they had a case of carcinoma of the rectum of a circumscribed nature which warranted an operation, and extirpation was performed, July 8, 1891. The patient did well up to the 16th of July, when a marked change came about rather unexpectedly during the day, ending in collapse. At 11 P. M. he died of septicæmia.

DR. J. W. HALLUM, of Carrollton, contributed a paper on

THE TREATMENT OF HEMORRHOIDS BY CARBOLIC ACID INJECTIONS.

In most of the case that apply for relief it is unnecessary to delay, but the speaker proceeds at once to make a radical cure of the pile tumor. He does this by mixing together pure carbolic acid one part, and pure glycerine two parts, and enough morphine and tannic acid that ten drops of mixture will contain one-quarter of a grain each. Let this be well shaken and thoroughly dissolved before using it.

The position that he has found to be probably the best is the one in which a toad assumes when in a sitting posture. In this position the patient will almost invariably pass out the tumors by an effort at straining down. There is more or less burning for a few minutes consequent from the injection, after which in the course of five or six hours the tumor begins to throb and ache from the swelling and also the death of the pile itself.

Adjourned.

---

THIRD DAY—AFTERNOON SESSION.

The following officers were elected for the ensuing year:

President, Dr. A. A. Smith, Hawkinsville; First Vice-President, Dr. Geo. J. Grimes, Columbus; Secoud Vice-President, Dr. R. H. Taylor, Griffin; Treasurer, Dr. C. E. Goodrich, Augusta; Secretary, Dr. D. H. Howell, Atlanta.

On motion, the Association adjourned to meet in Americus third Wednesday in April, 1893.

